# Multibreed genome wide association can improve precision of mapping causative variants underlying milk production in dairy cattle

**DOI:** 10.1186/1471-2164-15-62

**Published:** 2014-01-24

**Authors:** Lesley-Ann Raven, Benjamin G Cocks, Ben J Hayes

**Affiliations:** 1 Biosciences Research Division, Department of Primary Industries Victoria, 5 Ring Road, Bundoora 3086, Australia; 2 La Trobe University, Bundoora, Victoria 3086, Australia; 3 Dairy Futures Co-operative Research Centre, Bundoora, Victoria 3086, Australia

**Keywords:** Multibreed analysis, Genomic selection, Dairy cattle, Single nucleotide polymorphism

## Abstract

**Background:**

Genome wide association studies (GWAS) in most cattle breeds result in large genomic intervals of significant associations making it difficult to identify causal mutations. This is due to the extensive, low-level linkage disequilibrium within a cattle breed. As there is less linkage disequilibrium across breeds, multibreed GWAS may improve precision of causal variant mapping. Here we test this hypothesis in a Holstein and Jersey cattle data set with 17,925 individuals with records for production and functional traits and 632,003 SNP markers.

**Results:**

By using a cross validation strategy within the Holstein and Jersey data sets, we were able to identify and confirm a large number of QTL. As expected, the precision of mapping these QTL within the breeds was limited. In the multibreed analysis, we found that many loci were not segregating in both breeds. This was partly an artefact of power of the experiments, with the number of QTL shared between the breeds generally increasing with trait heritability. False discovery rates suggest that the multibreed analysis was less powerful than between breed analyses, in terms of how much genetic variance was explained by the detected QTL. However, the multibreed analysis could more accurately pinpoint the location of the well-described mutations affecting milk production such as *DGAT1.* Further, the significant SNP in the multibreed analysis were significantly enriched in genes regions, to a considerably greater extent than was observed in the single breed analyses. In addition, we have refined QTL on BTA5 and BTA19 to very small intervals and identified a small number of potential candidate genes in these, as well as in a number of other regions.

**Conclusion:**

Where QTL are segregating across breed, multibreed GWAS can refine these to reasonably small genomic intervals. However, such QTL appear to represent only a fraction of the genetic variation. Our results suggest a significant proportion of QTL affecting milk production segregate within rather than across breeds, at least for Holstein and Jersey cattle.

## Background

Variation in milk production traits and functional traits in dairy cattle have a major genetic component [1]. Genome wide association studies have been successful for identifying genomic regions which associate with these traits but few have led to identification of the underlying mutation (Eg. [2]). Within breed variation has been the focus of most analyses, however, within a cattle breed LD is extensive (eg r^2^ > 0.3 at 250 kb) making it difficult to map the mutation precisely, or even to a small number of candidate genes [3-7]. Between breeds however, linkage disequilibrium phase among SNP is only conserved at 5-10 kb, for *Bos taurus* breeds at least [7]. So, expanding cattle GWAS to multiple breeds could potentially refine QTL intervals [8].

This strategy has been used extensively in dogs [9]. The long distance LD in a single dog breed (r^2^ > 0.3 at 0.4-3.2 Mb) means that less markers are required for the initial association, but the precision of the mapping is poor [9]. To overcome this problem a two-stage mapping strategy is applied. A single breed with long distance LD is used to identify QTL, then multiple breeds and dense SNP typing are used for fine mapping [10]. As breeds can share ancestral mutations, recombination events can be used to refine QTL region [11]. The multibreed strategy allows mapping to a region of ~100 kb (reflecting the ancestral haplotype block size in multibreed dog populations) which typically contains a limited number of candidate genes.

Applying a multibreed strategy in dairy cattle may be useful to refine the location of QTL. It should be remembered, however, that while modern cattle breeds have been created relatively recently (< 400 generations, [12]), in some cases there has been strong selection since breed divergence [13]. As a result, some QTL segregating in one breed may not be segregating in the other. This study investigates the power of multibreed GWAS and explores the proportion of QTL segregating in multiple dairy cattle breeds, namely Holstein and Jersey dairy cattle. We hypothesise that a GWAS combining breeds will have more power to detect variation and that such GWAS should be able to map QTL to a smaller genomic interval. We conducted GWAS in 5240 Jerseys and 12685 Holsteins, both within breed and multibreed. Our results demonstrate that within breed analyses actually have more power to detect a higher proportion of the variation but multibreed GWAS results in the more precise mapping of the QTL that do segregate across breeds.

## Results

### Within breed analysis

To firstly determine how well we could map and validate QTL within breeds, we split the Holstein and Jersey data sets into cows and bulls within each breed (Additional file 1: Table S1 describes number of phenotypes for each trait in each data set). A GWAS was conducted using a mixed model including a regression on SNP genotype (0, 1 or 2 copies of the second allele), fixed effects of breed and gender, and a random polygenic breeding value effect to account for population structure. The significant SNP (P < 10^-8^) in the two genders within a breed were then compared.

For production traits, Holstein bulls and cows had approximately equal number of significant SNP except for protein where more SNP were significant in cows (Table 1), perhaps reflecting the greater range of phenotypes for this trait in the cows (Additional file 2: Table S2). Jersey cows and bulls differed in the number of SNP significant for all milk production traits with notably fewer SNP significant in Jersey bulls for fat, fat percentage and protein percentage. False discovery rates (FDR) were used to determine which group had more power to detect variation. FDRs were consistently low among milk production traits in all data sets (Additional file 3: Table S3). We then used a correlation of the common significant SNP to determine whether SNP effects went in the same direction in bulls and cows within breeds. Significant SNP shared between Holstein cows and Holstein bulls were highly positively correlated for all five milk production traits (Table 2). Correlation coefficients were lower between effects of significant SNP in Jersey bulls and cows for percentage traits (and significant SNP in Jerseys were mostly located on BTA14 and BTA20). Fewer SNP were shared for fat and fat percentage in Jerseys. To account for the possibility that different SNP were detecting the same QTL in the bulls and cows, we also investigated if for the cows there were significant SNP within 100 kb of the most significant SNP in the bulls and vice versa, however this did not greatly affect the number of SNP shared between Jersey cows and Jersey bulls (Table 1).

**Table 1:** Results of within breed and within gender genome wide association analysis

**Trait**	**Bulls**	**Cows**	**Overlapping**	**Number of 100 kb intervals containing SNP significant in both breeds**
*Jerseys*				
Fat	806	984	25 (3.10)	44 (4.0)
Milk	2117	3969	461 (21.8)	242 (23.5)
Protein	4016	2606	557 (13.9)	504 (24.1)
Fat%	677	5093	66 (9.75)	141 (16.1)
Protein%	692	5444	150 (21.7)	368 (34.2)
Fertility	16	1	0	0
Mamm. Syst.	1721	51	0	0
Survival	4302	206	1 (0.5)	10 (2.9)
SCC	248	3600	42 (16.9)	28 (19.0)
*Holsteins*				
Fat	2100	3899	1543 (73.5)	228 (82.0)
Milk	5321	5488	1529 (28.74)	1023 (31.6)
Protein	3288	8730	2936 (89.3)	178 (90.5)
Fat%	2084	3422	1353 (64.9)	372 (67.3)
Protein%	3378	4374	1644 (48.7)	1512 (54.1)
Fertility	1567	10	0	0
Mamm. Syst.	14770	147	28 (19.0)	79 (24.5)
Survival	3522	220	20 (9.1)	138 (14.5)
SCC	729	701	26 (3.7)	82 (7.0)

The number of SNP which are significant in both genders within Jerseys and Holsteins are presented (percentage of significant SNP that are significant in both genders given in parenthesis), as well as the number of 100 kb intervals containing significant SNP for that trait in both genders (percentage of 100 kb windows with a significant SNP in either gender with significant SNP in both genders given in parenthesis). Milk production traits were tested at P < 10^-8^ and reproductive and functional traits at P < 10^-5^.

**Table 2:** **Correlations of SNP effects for very significant SNP (P < 10**^**-20**^**) within genders within each breed, and between breeds for milk production, reproduction and health traits**

	**Bull/Cow**	**Hol/Jer**	**Holstein** **Bull/Cow**	**Bull/Cow** **Jersey**	
Fat	0.984	0.394	0.983	0.969	
Milk	0.973	0.082	0.978	0.973	
Protein	0.979	0.273	0.986	0.984	
Fat%	0.973	0.629	0.955	−0.057	
Protein%	0.977	0.034	0.965	−0.081	
Survival	0.979	0.997			
Mamm. Syst	0.979				
SCC	0.999				
Fertility	0.979				

For some functional traits, namely fertility (measured as calving interval), there were no SNP significant at P < 10^-8^. Therefore, we also considered a suggestive significance threshold of P < 10^-5^ for these traits. In Holsteins, there were fewer SNP significant for SCC and Holstein cows displayed far fewer significant associations than Holstein bulls, which was also reflected in higher FDRs (Table 1; Additional file 3: Table S3). Jersey bulls showed a much larger number of significant SNP for the functional traits than Jersey cows (Table 1). Few SNP were significant for fertility and Jersey cows had many more significant SNP for SCC than Jersey bulls. There were few SNP shared within breeds for fertility and mammary system in Jerseys and fertility within Holsteins (Table 1). Although a much lower significance threshold was used, the proportion of common SNP for mammary system, survival and SCC still did not reach the levels observed for milk production traits.

The observation that fewer SNP are shared between cows and bulls for Jerseys than for Holsteins is likely a reflection of smaller sample size. Further, for non-production traits, the lower heritability of these traits likely leads to lower power, in both the Holstein and the Jersey analysis, and particularly for cows. There was a much larger number of cows in the analysis (Additional file 1: Table S1) but more accuracy associated with the phenotype for the bulls, which are daughter trait deviations from thousands of daughters in some cases. There were especially large variations in the number of significant SNP between genders for mammary system and survival.

### Comparison of QTL between breeds

The cow and bull data sets were combined within breed and GWAS performed. Results were then compared between the breeds. For milk production traits, some SNP were significant in both breeds, however, Holsteins showed more significant SNP for all traits except fat and fat% (Table 3). We identified very significant milk production QTL (P < 10^-20^) on BTA5, BTA14 and BTA20 in both breeds (Additional file 4: Table S4). For Holsteins, we also identified very significant QTL on BTA26 for fat, BTA6 for fat percentage and protein percentage and BTA29 for protein percentage. Very significant QTL were observed in Jerseys, but not for Holsteins, on BTA17 for fat, BTA19 for protein percentage and BTA3, BTA10 and BTA29 for fat percentage. Smaller protein and milk QTL were distributed throughout most chromosomes. The proportion of significant SNP shared between the breeds was less than 8% for all traits except fat percentage and protein percentage which had 38.96% and 27.33% respectively (Table 3). To account for the fact that LD phase may be different between the breeds, and breeds exhibit variation in LD patterns, we searched for significant SNP (P < 10^-8^) within a 100 kb window of any SNP that was significant in one breed but not the other (i.e. those not common to both breeds). This increased the proportion of SNP shared by 3-8%. There was a moderate positive correlation in the effects of the SNP for significant SNP associating with fat percentage in Holstein and Jerseys (Table 2).

**Table 3:** Results of within breed genome wide association analysis

**Trait**	**Holsteins**	**Jerseys**	**Overlapping**	**Number of 100 kbintervals containing SNP significant in both breeds**
Fat	2654	5718	124 (4.7)	633 (7.5)
Milk	23808	17573	1210 (6.9)	6341(14.2)
Protein	33671	21065	1327 (6.3)	8804 (14.9)
Fat%	7688	12141	2996 (38.9)	2416 (43.1)
Protein%	12141	7434	2032 (27.3)	3243 (33.4)
Fertility	2276	125	0	1 (0.8)
Mamm. Sys	12088	3032	130 (4.3)	707 (11.1)
Survival	3930	6263	300 (7.6)	739 (12.3)
SCC	1693	3507	36 (2.1)	101 (6.1)

The number of SNP which are significant in both breeds are presented (percentage of significant SNP that are significant in both breeds given in parenthesis), as well as the number of 100 kb intervals containing significant SNP for that trait in both breeds (percentage of 100 kb windows with a significant SNP in either breed with significant SNP in both breeds given in parenthesis). Milk production traits were tested at P < 10^-8^ and reproductive and functional traits at P < 10^-5^.

Fewer significant associations were found for health and reproductive traits in either breed. However, for mammary system, a number of significant associations were detected in Holsteins and also for survival in Jerseys (Table 3). Health and reproductive traits were significant at P < 10^-8^ across many chromosomes, however at P < 10^-20^ we were only able to identify major QTL for BTA18 for survival, BTA11 for mammary system and BTA10 for SCC in Holsteins. Jerseys showed very significant QTL on BTA2, BTA6, BTA11 and BTA25 for survival. False discovery rates were very high for fertility, particularly in Jerseys (Additional file 3: Table S3). Estimates for survival and SCC appear less powerful than milk production traits with moderate error rates, again likely reflecting lower heritabilities for these traits. When the suggestive threshold was used (P < 10^-5^) there were still few SNP common between Holsteins and Jerseys for these traits. Where significant SNP were common to each breed, we used a correlation analysis to determine whether the direction of effects was the same in both breeds. We identified a strong positive correlation between the common SNP significantly associating with survival, however, there were few significant SNP in the comparison, so sample size may be inadequate to affirm this (Table 2).

### Multibreed analysis

We performed a multibreed GWAS using all the combined Holstein and Jersey data to investigate the potential of such analyses to refine confidence intervals. In the multibreed GWAS, very significant QTL (P < 10^-20^) were identified for all milk production traits (Table 4; Additional file 4: Table S4). A number of QTL were located within several previously described genomic regions. These QTL regions typically had effects on a number of the milk production traits. Significant associations (P < 10^-8^) were identified for all health traits but not fertility (Additional file 4: Table S4). Of the non-production traits, very significant QTL (P < 10^-20^) were only identified for survival.

**Table 4:** Most significant SNP regions and candidate genes for production and functional traits in dairy cattle from a large multibreed GWAS

**Trait**	**SNP name**	**Chr**	**Position (bp)**	**log(10)P**	**Within Gene**	**100 kb LD**	**<500 kb LD**	**Gene name**
**Fat**	ARS-BFGL-NGS-4939	14	1801116	255.551	DGAT1			diacylglycerol O-acyltransferase 1
	BTB-00932332	26	22118554	24.142	BRTC			Beta-transducin repeat containing
	BovineHD0500026662	5	93945655	23.718		MGST1		microsomal glutathione S-transferase 1
	BovineHD1800017481	18	60506726	12.907	LOC788871			zinc finger protein 85-like
	BovineHD1200015001	12	54273515	12.918			RNF219	ring finger protein 219
**Milk**	ARS-BFGL-NGS-4939	14	1801116	416.491	DGAT1			diacylglycerol O-acyltransferase 1
	BovineHD2000009925	20	34582764	44.167			LOC782462	sorting nexin-13-like
	BovineHD0500026852	5	94562606	24.636	EPS8			epidermal growth factor receptor pathway substrate 8
	BovineHD0600024338	6	88865430	22.814			GC	group-specific component (vitamin D binding protein)
	BovineHD4100003579	5	32784231	19.562	RPAP3			RNA polymerase II associated protein 3
**Protein**	ARS-BFGL-NGS-4939	14	1801116	144.166	DGAT1			diacylglycerol O-acyltransferase 1
	BovineHD4100005296	6	87180731	31.840	CSN2			casein beta
	BovineHD2600004009	26	15654751	21.778	PLCE1			phospholipase C, epsilon 1
	BovineHD0500029843	5	104307736	19.303	CD27			CD27 molecule
	BovineHD2600011254	26	40809501	18.214			PPAPDC1A	phosphatidic acid phosphatase type 2 domain containing 1A
**Fat%**	ARS-BFGL-NGS-4939	14	1801116	1684.117	DGAT1			diacylglycerol O-acyltransferase 1
	BovineHD0500026662	5	93945655	93.370		MGST1		microsomal glutathione S-transferase 1
	BovineHD2000009925	20	34582764	61.199			LOC782462	sorting nexin-13-like
	ARS-BFGL-NGS-57448	27	36155097	32.124	GINS4			GINS complex subunit 4 (Sld5 homolog)
	BovineHD0200034371	2	119076939	13.851		SP110		SP110 nuclear body protein
**Protein%**	ARS-BFGL-NGS-4939	14	1801116	398.865	DGAT1			diacylglycerol O-acyltransferase 1
	BovineHD2000009927	20	34587828	180.352			LOC782462	sorting nexin-13-like
	BovineHD0600023879	6	87160102	70.809		CAS1A		casein alpha s1
	BovineHD0300005054	3	15451257	44.404	GBA			glucosidase, beta, acid
	BovineHD4100003579	5	32784231	34.001	RPAP3			RNA polymerase II associated protein 3
**Fertility**	BovineHD1800016761	18	57548213	7.445	LOC786539			carcinoembryonic antigen-related cell adhesion molecule 18-like
	BovineHD0600024357	6	88922396	6.712			NPFFR2	neuropeptide FF receptor 2
	BovineHD0500024481	5	86451512	6.441			SOX5	SRY (sex determining region Y)-box 5
	BovineHD0400015668	4	57384888	6.371			LOC100298628	HIG1 domain family, member 1D-like pseudogene
	BovineHD0100005095	1	16862494	6.215	LOC100140852			magnesium transporter NIPA2-like
**Mamm. System**	BovineHD2600006024	26	23379482	9.478	SUFU			suppressor of fused homolog (Drosophila)
	BovineHD0200030267	2	105149497	8.263	SMARCAL1			SWI/SNF related, matrix assoc., actin dep. Reg. of chromatin, subfamily a-like 1
	BovineHD1100008368	11	28171549	8.175	PRKCE			protein kinase C, epsilon
	BovineHD0200035718	2	123208383	8.069		SDC3		syndecan 3
	BovineHD2000008146	20	27565339	7.890			ISL1	ISL LIM homeobox 1
**Survival**	BovineHD1100031193	11	107227039	67.300		NLRP6		NLR family, pyrin domain containing 6
	Hapmap40387-BTA-107848	9	104960154	35.305		WDR27		WD repeat domain 27
	BovineHD3000041679	30	144726003	33.731			TBL1X	transducin (beta)-like 1X-linked
	BovineHD2000006692	20	22290945	31.258		MIER3		mesoderm induction early response 1, family member 3-like
	BovineHD1700000492	17	2237102	30.497			NPY2R	neuropeptide Y receptor Y2
**SCC**	ARS-BFGL-NGS-92033	1	2582894	12.503		MIS18A		MIS18 kinetochore protein homolog A (S. pombe)
	ARS-BFGL-NGS-57102	3	31219298	10.716		CTTNBP2NL		CTTNBP2 N-terminal like
	BTA-104934-no-rs	7	67940770	10.324	LARP1			La ribonucleoprotein domain family, member 1
	BovineHD1100009904	11	32859722	9.169	NRXN1			neurexin 1
	BovineHD1600008712	16	30646220	8.556		ADCK3		aarF domain containing kinase 3

Interestingly, in the multibreed analysis there were more significant SNP located within genes than in single breed analysis (Table 5). While enrichment was not overwhelming and did not hold across the functional traits, it does appear that we are closer to refining QTL using multibreed data, at least in the milk production traits.

**Table 5:** **Comparison of the proportion of SNP occurring within genes in the whole genome (632 K SNP) and SNP significant (P < 10**^**-8**^**) in the multibreed and single breed analyses**

	**Proportion of SNP Within Genes**			
	**632 K SNP**	**Multibreed (P < 10**^**-8**^**)**	**Holsteins (P < 10**^**-8**^**)**	**Jerseys (P < 10**^**-8**^**)**
**Fat**	0.319	0.381	0.361	0.324
**Milk**	0.319	0.348	0.330	0.323
**Protein**	0.319	0.363	0.329	0.327
**Fat%**	0.319	0.374	0.356	0.332
**Protein%**	0.319	0.327	0.332	0.311
**SCC**	0.319	0.375	0.200	0.399
**Survival**	0.319	0.296	0.344	0.392
**Mamm D**	0.319	0.150	0.328	0.191

**Figure 1:**
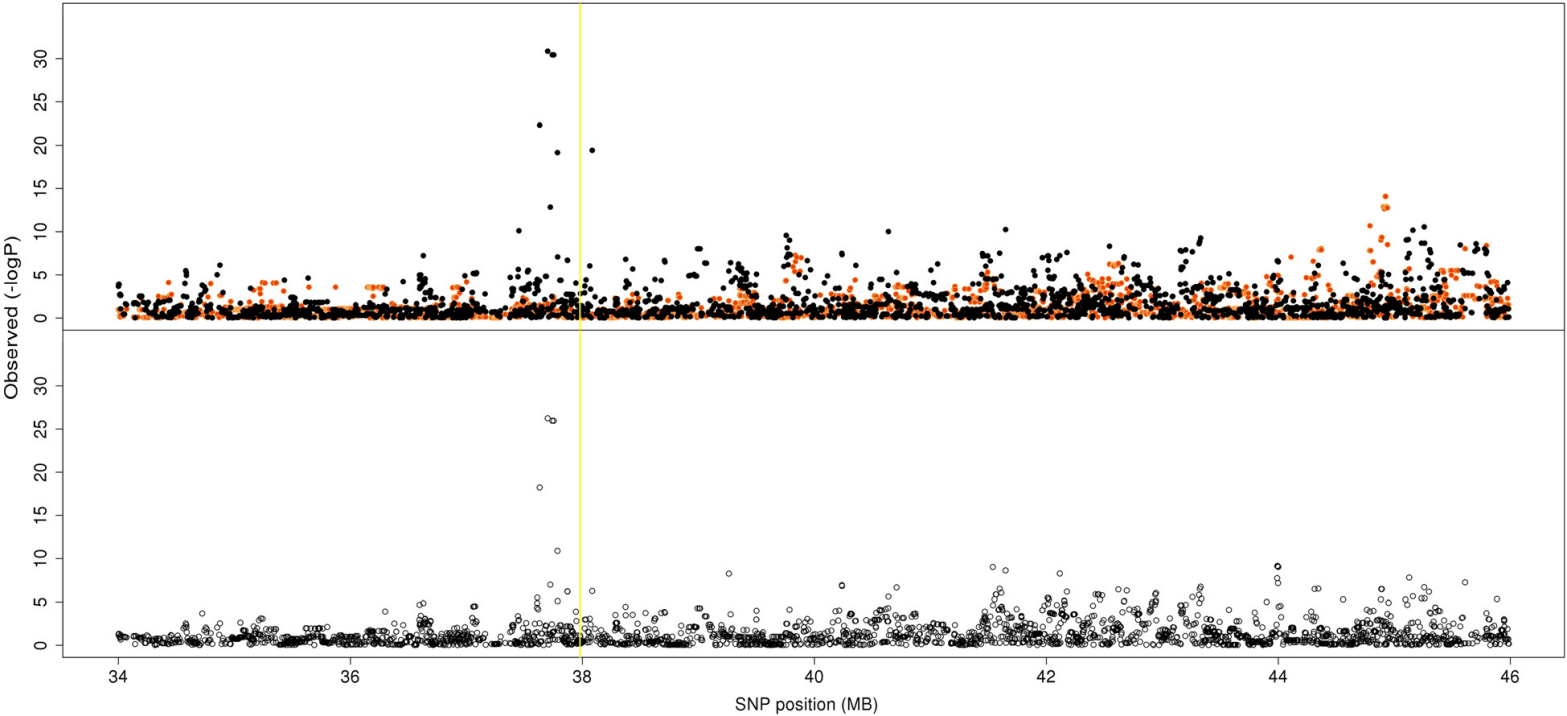
**Association analysis of SNP surrounding *GHR *on BTA20.** Associations of SNP in a 30 Mb region of BTA20 with protein percentage in Holsteins (Black dots), Jerseys (Orange dots) and in a multibreed GWAS (lower panel). The yellow vertical line represents the position of the *GHR* gene.

False discovery rates were consistently low for all milk production traits and survival suggesting that the multibreed GWAS is powerful for detecting associations and minimising errors (Additional file 3: Table S3). However, FDRs were higher than in the between breed analyses, and there were a smaller number of QTL regions significant at P < 10^-8^ than the between breed GWAS (Additional file 4: Table S4). Breed differences are quite clear in some cases; for example, when plotting the *GHR* region of BTA20 the QTL is not segregating in Holsteins or Jerseys (Figure 1). Within breeds there were less very significant QTL identified (P < 10^-20^) than in the multibreed analysis (Additional file 4: Table S4). For QTL segregating in both breeds, the multibreed analysis does seem to increase power.

**Figure 2:**
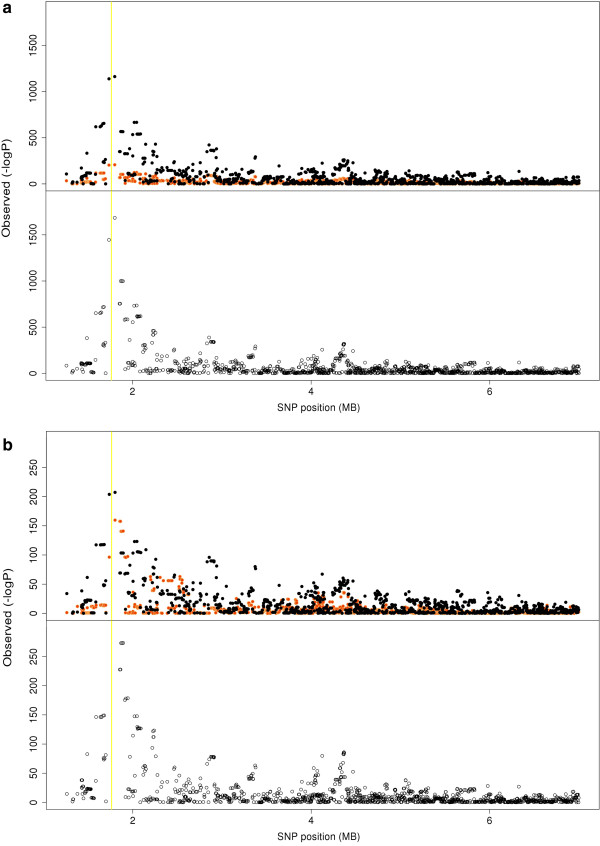
**Association analysis of SNP surrounding *ABCG2 *on BTA6.** Associations of SNP in a 12 Mb region of BTA6 with protein percentage in Holsteins (Black dots), Jerseys (Orange dots) and in a multibreed GWAS (lower panel). The yellow vertical line represents the position of the *ABCG2* gene.

**Figure 3:**
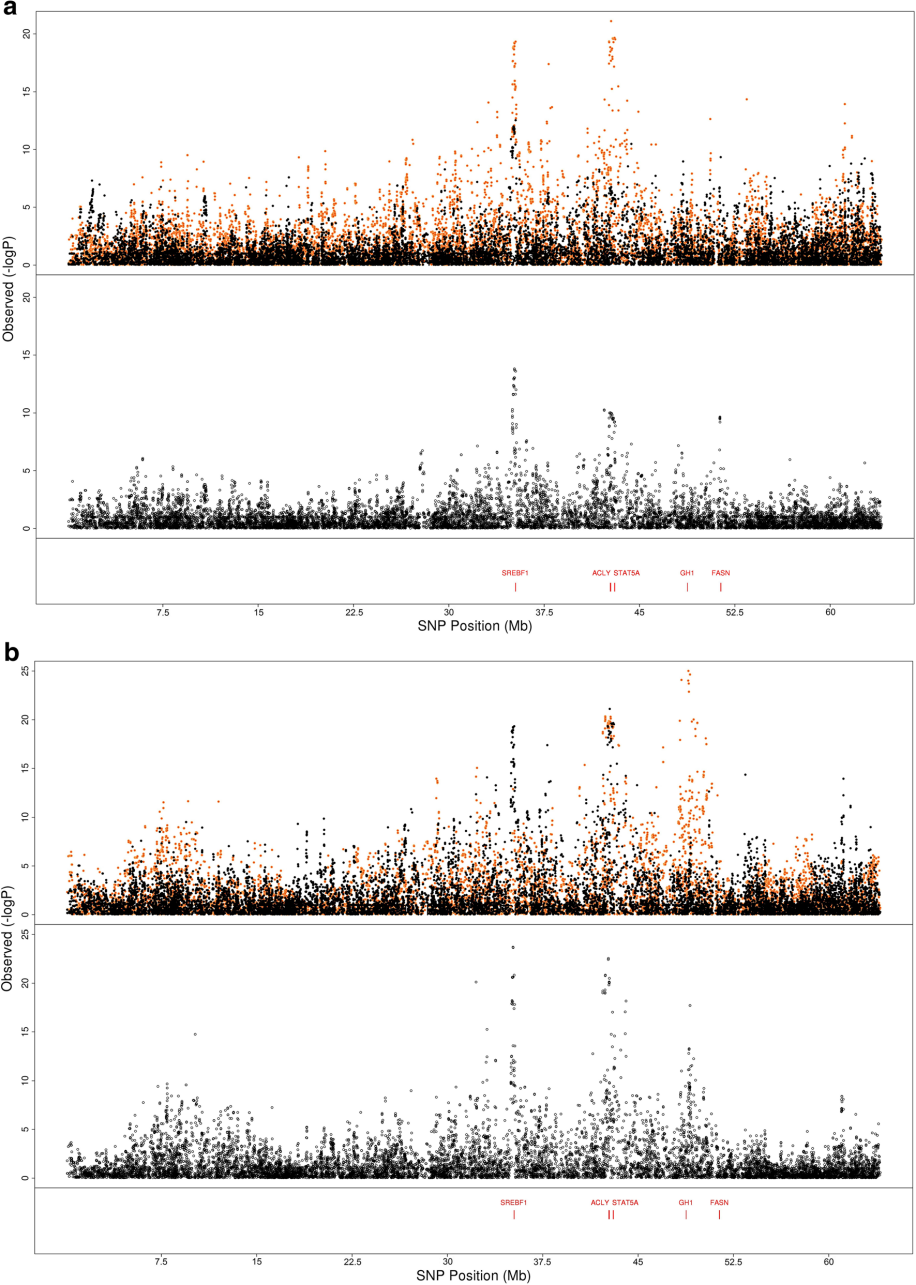
**Association analysis of SNP surrounding *DGAT1 *on BTA14.** Associations of SNP in a 7 Mb region of BTA14 for **a)** fat percentage and **b)** protein percentage for Holsteins (Black dots), Jerseys (Orange dots) and in a multibreed GWAS (lower panel). The yellow vertical line represents the position of the *DGAT1* gene.

Known QTL were used to determine whether the multibreed GWAS was more effective at narrowing QTL regions than single breed models. SNPs in the region surrounding the *ABCG2* locus [14] on BTA6 showed a much stronger signal in Holstein within breed analysis than the multibreed analysis (Figure 2), as there was no evidence that this mutation was segregating in our Jersey population. Hence, the multibreed analysis would not refine the QTL interval in this case. The putative QTL at *GHR* gene on BTA20 was also mapped (Figure 1). Our results, and those of others, strongly suggest that *GHR* may not be the associated gene in this region [15]. There most significant SNP are 1 Mb to the right of *GHR*. This peak at least is somewhat sharper in the multibreed analysis. Several genes may be associated in this region, as there is another peak at approximately 36 Mb, and the multibreed analysis does appear to resolve these peaks to somewhat smaller intervals. For both fat percentage and protein percentage, the Holstein QTL was much more significant at *DGAT1* than the Jersey QTL (Figures 3a and 3b). The multibreed peak was very slightly closer to *DGAT1* than when breeds were considered separately (~80,000 bp), and the level of significance was considerably higher for both traits. So for large QTL that segregate in multiple breeds, there, appears to be more precision in the multi-breed analysis.

**Figure 4:**
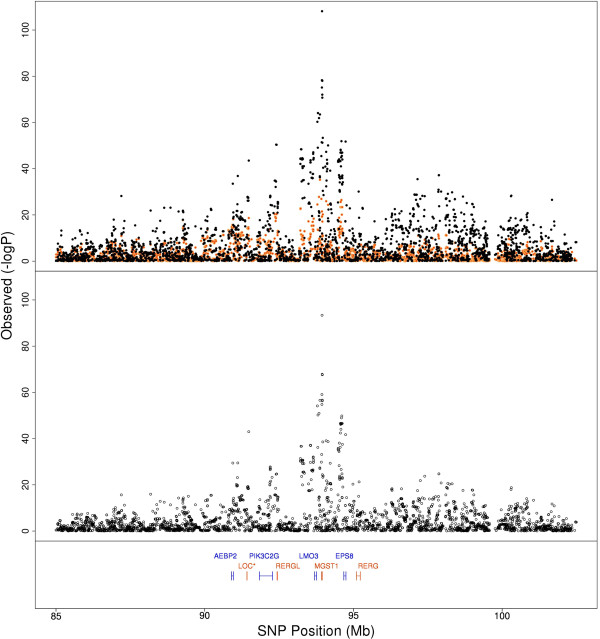
**Association analysis of SNP on BTA19.** Associations of SNP on BTA19 encompassing a cluster of fat metabolism genes for **a)** fat percentage and **b)** protein percentage in Holsteins (black dots, upper figure), Jerseys (orange dots, upper figure), and in a multibreed population (lower panel of each Figure).

**Figure 5:**
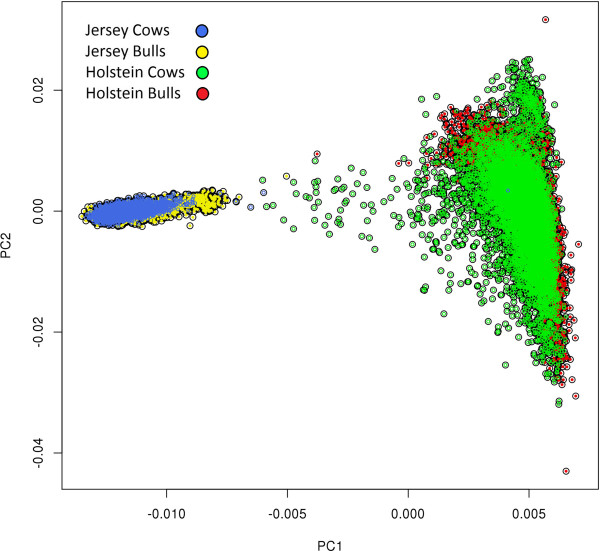
**Association of SNP on BTA5.** Associations of SNP in a 20 Mb QTL region of BTA5 with fat percentage in Holsteins (black dots, upper figure), Jerseys (orange dots, upper figure) and in a multibreed population (lower panel).

We identified a highly significant fat percentage and protein percentage QTL at 42.7 Mb on BTA19. The most significant SNP in this region sits within *ACLY*, a fatty acid biosynthesis gene [16] (Figures 4a and 4b). Previously, *ACLY* was described within this region along with several other fat metabolism genes *FASN*, *GH*, *SREPB1* and *STAT5A* but there has been little power to refine this region [17]. In the multibreed analysis, we can actually identify separate peaks for *SREBP1*, *FASN* and *GH* (Figures 4a and 4b). However, *STAT5A* lies only 300 kb upstream of *ACLY* so we cannot rule out *STAT5A* as potentially harbouring a causal mutation associated with these significant SNP. There was another peak on BTA5, at 85-110 Mb which showed highly significant QTL across all milk production traits. The top SNP of two close peaks localised to within 3000 bp of *MGST1* and to within *EPS8* (Figure 5). The significant SNP corresponding to *MGST1* and *EPS8* sit 600 kb apart on BTA5 and appear to be individual QTL, as the r^2^ between the SNP in these genes is very low (The r^2^ between the most significant SNPs in the two genes is only 0.06, so it is unlikely that they are picking up the same QTL).

## Discussion

We performed a GWAS in a large multibreed dairy cow population with 632,003 SNP to identify the genomic regions associating with milk production, health and reproduction traits. We aimed to determine whether multibreed data sets are more powerful and result in more precise mapping of QTL. The results suggest that the multibreed analysis is actually less powerful in terms of number of QTLs identified, as there are a considerable number of QTL that only segregate in one breed. However, when QTL did segregate across breeds, the multibreed analysis refined QTL to smaller genomic regions, allowing a small number of potential candidates to be identified.

**Figure 6:**
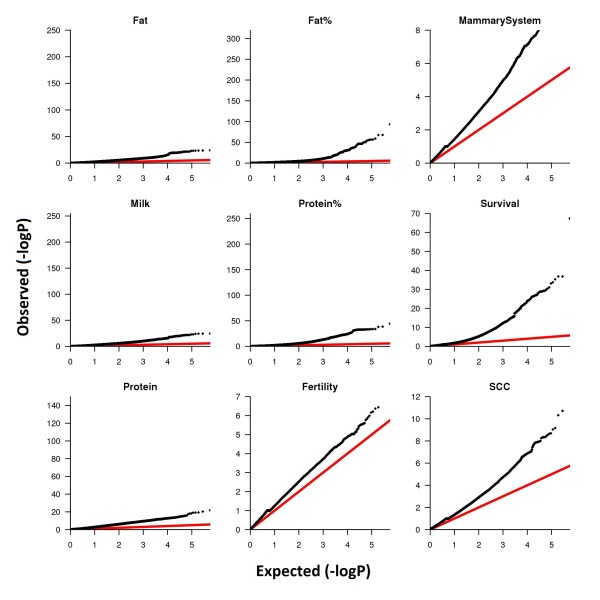
Principal component analysis of the genomic relationship matrix, with animals coloured by breed and sex.

Our results suggest that a significant proportion of QTL segregate only within one breed or the other. Combining cows and bulls provided more power to identify more QTL affecting the traits than separating the sexes within breed, however combining breeds did not. Holstein and Jersey cattle showed obvious genetic differentiation in a principle components analysis (Figure 6, a principal component analysis of genetic diversity), thus it was perhaps not surprising that all QTL were not shared between Holsteins and Jerseys. However, the proportion of QTL that we observe to be shared between breeds will be affected by the power of the experiments. The effect of power is demonstrated by the different proportion of QTL shared for traits within different heritabilities. Generally, those with the highest heritabilities (protein percentage and fat percentage) had the largest number of shared QTL while those traits with the lowest heritabilties had the lowest numbers of shared QTL (Table 3).

Results for functional traits were inconsistent within and between breeds. For example, there was a limited overlap of significant QTL within the Jersey breed. Lower heritabilities (in particular larger environmental variation) reduced power for these traits particularly in Jerseys and in cows of both breeds. We observed very few QTL that segregated across breeds for these traits. Again, this is likely an artefact of the low power for these traits. Another possible explanation would be strong selection for these traits has fixed QTL in one breed and not the other. The common significant SNP shared within Holsteins (between bulls and cows) were highly positively correlated, in effect and in direction. This suggests that sample size may be more important than gender differences in power. This was previously shown in beef breeds where using breeds with smaller sample sizes reduced the power to identify segregating loci [18].

Very significant QTL (P < 10^-20^) were identified in both the multibreed analysis, and the within breed analyses. Among these were several QTL that had been previously described. SNP close to *DGAT1* were highly associated for all milk production traits in both breeds. A very significant QTL on BTA20 has been previously ascribed to a mutation in *GHR*[15,19], but is more likely a number of separate QTL given the results of our multibreed analysis. Another very significant QTL was identified on BTA14 at approximately 70Mb for fat percentage, protein percentage and milk. This QTL appears to locate around the *SDC2* gene. Previous studies have shown evidence of QTL at the telomeric end of BTA14 but this was not confirmed here [20]. We also identified a major QTL on BTA6 for protein kg, which centres within *CSN1*. For protein kg, a major QTL on BTA5 centres within *CD27*.

The value of the multibreed analysis was highlighted by the fact that more SNP were located within genes than when breeds were separated, suggesting there is more precision in refining QTL in the multibreed analysis. Inspection of several QTL regions support this. For example, a region on BTA19 was refined such that a strong candidate, the lipid metabolism gene *ACLY*, could be identified. *ACLY* is differentially express in rat mammary tissue during pregnancy and lactation [21]. This region of BTA19 was identified in a GWAS phenotyping the ratio of saturated to unsaturated fats in the milk of Danish Holstein cows but is otherwise undescribed [17]. The multibreed analysis shows a clear benefit over breed dependent methods in refining the precise locations of these fat metabolism genes.

The very significant QTL affecting fat kg and fat percentage on BTA5 centred within 3000 bp of *MGST1*, an inflammation response gene which is highly expressed through pregnancy and lactation [22]. *MGST1* was upregulated during adipocyte development in the *Longissimus* muscle in Japanese Black cattle [23]. Slightly upstream, a highly significant peak centred within *EPS8. EPS8* acts as a receptor tyrosine kinase substrate for epidermal growth factor receptor (EGFR) and thus increases the signalling response to epidermal growth factor (EGF) [24]. A previous study on German Holsteins identified *EPS8* as a candidate fat percentage gene [25]. Another study found a milk yield QTL from 92.1-93 Mb but did not localise to near *MGST1*[26]. Our results suggest both *MGST1* and *EPS8* may contain QTL affecting milk production.

Other QTL have been identified at the telomeric end of this chromosome which do not align with our results. Cole *et al.* identified fat yield QTL at 98.7 Mb, but our peak was 1 Mb downstream within *LOH12CR1*[1]. A 50 k GWAS of Canadian Holsteins identified several fat yield candidate genes including *SLC2A3* and *LOC786521* (GDF3) at 101.7-101.8 Mb and *LRP6*, *LOC786490* (EMP1) and *DUSP16* at 97-98 Mb [27]. This region was highly significant for milk and protein in our study rather than fat and their candidate genes do not lie within our QTL peak.

A limitation of this study is the use of a set of ascertained SNP to map QTL. Interpretations regarding the position of QTL which segregate across breeds must be treated with caution, as this will depend on the extent of linkage disequilibrium between breeds with causative mutations. As whole genome sequence data becomes increasingly available, this study could be replicated using imputed, full-sequence genotypes to determine whether the Holsteins and Jerseys still maintain a larger proportion of significant variants than the multibreed sample.

Finally, population stratification is a key cause of false positive results in GWAS as admixture occurs subtly in the form of relationships among animals [8]. In our analysis, where breeds are combined, we have attempted to account for stratification both by fitting a breed effect and pedigree effect within breed. As previously seen (and in QQ plots, Additional file 5: Figure S1), our within breed analysis actually reduced the number of significant effects suggesting combining the breeds is not leading to an increase in the number of false positives [6,28]. Finally, multibreed GWAS is likely to identify older, conserved mutations but may not be as effective as a single breed model for recently diverged mutations.

## Conclusion

A multibreed analysis together with dense SNP genotypes has allowed us to refine QTL locations for milk production and functional traits – for example this approach allowed us to refined QTL on BTA5 and BTA19 to a limited number of candidate genes. Further evidence that the multibreed analysis refines QTL regions is that we observed an enrichment of significant SNP within genes in the multibreed analysis. However, there is still a considerable role for studies on individual breeds as our results suggest a considerable proportion of QTL do not segregate across breeds (for Holstein and Jersey cattle at least). In future, using sequence data rather than SNP array genotypes combined with a multibreed analysis could potentially lead to direct identification of the causative mutation.

## Methods

### Phenotypes and genotypes

There were 17925 dairy cattle in the study in total. Of these 9289 were Holstein cows, 3396 were Holstein bulls, 4226 were Jersey Cows and 1014 were Jersey Bulls (Additional file 1: Table S1). Phenotype records were available for a range of milk production, health and reproductive traits including milk yield, fat yield, protein yield, fat%, protein%, protein kg, fertility (calving interval), mammary system, somatic cell count and survival [29,30]. The phenotypes were trait deviations for cows and daughter yield deviations for bulls. Trait deviations were calculated from raw phenotypes in a very large data set of approximately one million cows, fitting a model which included herd year season, age of cow and permanent environment effect. Daughter trait deviations for bulls were then calculated from the trait deviations of their daughters, corrected for breed of mate. Records were standardised to have a mean of zero and standard deviation of one in both breeds.

Each animal was SNP genotyped either using the Illumina BovineHD BeadChip or the Illumina 50 k Bovine chip. Filtering of SNP was performed as described by [31]. Animals genotyped for 50 k were imputed to HD genotypes as described by Erbe, Hayes et al. (2012). Map positions of these SNP (and candidate genes) were from the Bovine Genome UMD3.1 assembly in the NCBI database (NCBI, http://www.ncbi.nlm.nih.gov)[32].

Animals were divided into subsets for analysis (Additional file 1: Table S1). First, the sample was divided by gender within breed to create a dataset for within breed comparisons. To compare the proportion of significant SNP for each trait for each breed, cows and bulls within each breed were analysed together. Finally, the entire sample of 17925 animals was used for a full multibreed analysis (Additional file 1: Table S1).

### Association analysis

A mixed model including a regression on the number of second alleles was fitted using ASReml [33]. The linear mixed model was

y=μ+Xβ+Ζu=e

where **y** is a vector of phenotypes (DTDs for bulls and TDs for cows. In all analyses, bull phenotypes were weighted following Garrick et al. [34].

$$\frac{1-h^{2}}{\left[\frac{1+\left(4-h^{2}\right)}{n}\right]}$$

where ***n*** represents the number of daughters. Cow phenotypes were weighted using the formula

$$\frac{1-h^{2}}{\left[\frac{1+r^{2}\left(n-1\right)}{n}\right]}$$

Where ***r***^**2**^ is the repeatability and ***n*** is the number of observations (e.g. lactations). For the percentage traits and survival in the bulls we were unable to fit weights in the model due to convergence problems). Other terms in the model were μ**,** the mean, **X** is the vector of animal genotypes (0,1or 2 copies of the second allele), β is the SNP effect, **Z** is the incidence matrix mapping phenotype to animal, **u** is the vector of polygenic effects and **e** is the vector of random residuals. The polygenic breeding values were fitted to control for population structure as random effects following a normal distribution $N\left(0,A\sigma_{a}^{2}\right)$ where **A** is the expected relationship among individuals constructed from the pedigree (which dated back to the 1940s) and $\sigma_{a}^{2}$ is the polygenic genetic variance (e.g. [35]). Significant SNP were selected at P-value thresholds of P ≤ 10^-8^ for milk production traits, SCC and survival. As no SNP were significant at this threshold for fertility, a suggestive threshold of P ≤ 10^-5^ was also used for fertility and other functional traits. We calculated the false discovery rate (FDR) for our GWAS at P ≤ 10^-8^ and P ≤ 10^-5^. FDR was defined as

$$m\cdot \frac{P}{S}$$

where, *m* is the number of tests, P is the probability value of the F-test and S is the proportion of significant SNP [36]. A Q-Q plot showing the distribution of significant effects is provided (Additional file 5: Figure S1).

For Holstein cows and Holstein bulls and Jersey cows and Jersey bulls we assessed how many of the significant SNP were the same in both GWAS. We then expanded to 100 kb either side of the SNP in order to account for the possibility that the same causative mutation may be in LD with a different SNP in the different breeds. We also correlated the effects of these SNP for the two breeds to determine if the SNP were in the same linkage phase with the QTL. We also investigated genes reported to have mutations affecting milk production, namely *DGAT1*, *GHR* and *ABCG2* , to further describe the precision of mapping within breeds and in the multibreed analysis for refining QTL position [14,19,37].

### Availability and requirements

A full SNP map and P-values for 9 traits are provided in Additional file 6. Programs, scripts and information for setting up the analysis can be obtained from the authors upon request.

### Ethics statement

There were no animal studies conducted for this manuscript. Where animal data was used references have been provided.

## Competing interests

The authors declare that there are no conflicting interests.

## Authors’ contributions

LR performed all analyses and drafted the manuscript. BC and BH conceived the study and participated in the design and coordination. All authors read and approved the manuscript.

## Supplementary Material

Additional file 1: Table S1 Number of phenotypes for production and functional traits in the data set. Click here for file

Additional file 2: Table S2 The minimum and maximum phenotypes for production and functional traits in dairy cattle. Phenotypes are expressed in standard deviations, with a mean of zero within each breed. Click here for file

Additional file 3: Table S3 False discovery rates for comparative GWAS at a) P < 10^-8^ significance and b) a suggestive threshold of P < 10^-5^. (NS) No significant SNP to test. Click here for file

Additional file 4: Table S4 The number of very significant QTL (P < 10^-20^) identified for milk production traits and significant QTL (P < 10^-8^) for production and functional traits, within breeds, within genders, and in the multibreed data set. Click here for file

Additional file 5: Figure S1 Q-Q Plot of significant effects for milk production traits in Holstein and Jersey cattle. (a) The large number of significant SNP in the milk production trait plots are driven by a small number of QTL regions, as demonstrated by re-drawing the plots with BTA6, BTA14, and BTA20 removed from the analysis (b). Click here for file

Additional file 6 SNP map and P-values for multibreed GWAS of 9 milk production traits. Click here for file
