# Supplementary material for: Multibreed genome wide association can improve precision of mapping causative variants underlying milk production in dairy cattle
Source: BMC Genomics. 2014 Jan 24;15:62. doi: 10.1186/1471-2164-15-62 (PMC3905911; doi:10.1186/1471-2164-15-62)
Supplement: Additional file 1: Table S1 — Number of phenotypes for production and functional traits in the data set. [file 1471-2164-15-62-S1.doc]

**Additional file 1: Table S1. Number of phenotypes for production and functional traits in the data set.**

| **Phenotype** | **Multibreed analysis** | **Between breed analysis** | | **Within breed analysis** | | | |
| --- | --- | --- | --- | --- | --- | --- | --- |
|  |  | **Jerseys** | **Holsteins** | **Hol Cow** | **Hol Bull** | **Jer Cow** | **Jer Bull** |
| **Fat** | 16812 | 5027 | 11785 | 9015 | 2770 | 4202 | 825 |
| **Milk** | 16812 | 5027 | 11785 | 9015 | 2770 | 4202 | 825 |
| **Protein** | 16812 | 5027 | 11785 | 9015 | 2770 | 4202 | 825 |
| **Fat %** | 16207 | 4755 | 11452 | 8718 | 2734 | 3948 | 807 |
| **Protein %** | 16812 | 5027 | 11785 | 9015 | 2770 | 4202 | 825 |
| **Fertility** | 15430 | 4732 | 10698 | 8135 | 2563 | 4009 | 723 |
| **Mamm. Sys** | 6910 | 2292 | 4618 | 2882 | 1736 | 1874 | 418 |
| **Survival** | 15352 | 4922 | 10430 | 7775 | 2655 | 4143 | 779 |
| **SCC** | 16297 | 4919 | 11378 | 8752 | 2626 | 4175 | 744 |
