# Supplementary material for: Multibreed genome wide association can improve precision of mapping causative variants underlying milk production in dairy cattle
Source: BMC Genomics. 2014 Jan 24;15:62. doi: 10.1186/1471-2164-15-62 (PMC3905911; doi:10.1186/1471-2164-15-62)
Supplement: Additional file 2: Table S2 — The minimum and maximum phenotypes for production and functional traits in dairy cattle. Phenotypes are expressed in standard deviations, with a mean of zero within each breed. [file 1471-2164-15-62-S2.doc]

**Additional file 2: Table S2. The minimum and maximum phenotypes for production and functional traits in dairy cattle. Phenotypes are expressed in standard deviations, with a mean of zero within each breed.**

|  | **Multibreed** | | **Holsteins** | | **Jerseys** | | **Hol Cow** | | **Hol Bull** | | **Jer Cow** | | **Jer Bull** | |
| --- | --- | --- | --- | --- | --- | --- | --- | --- | --- | --- | --- | --- | --- | --- |
|  | **Min** | **Max** | **Min** | **Max** | **Min** | **Max** | **Min** | **Max** | **Min** | **Max** | **Min** | **Max** | **Min** | **Max** |
| **Fat** | -4.315 | 4.815 | -4.315 | 4.811 | -3.483 | 4.815 | -4.315 | 4.811 | -2.804 | 2.216 | -3.273 | 4.815 | -3.483 | 1.633 |
| **Milk** | -4.804 | 5.555 | -4.804 | 5.555 | -3.327 | 3.997 | -4.804 | 5.555 | -2.499 | 2.946 | -3.327 | 3.997 | -3.261 | 2.110 |
| **Protein** | -5.441 | 5.249 | -5.441 | 5.249 | -4.214 | 4.502 | -5.441 | 5.249 | -2.863 | 2.660 | -4.214 | 4.502 | -3.837 | 1.925 |
| **Fat %** | -0.280 | 0.207 | -0.280 | 0.207 | -0.240 | 0.174 | -0.280 | 0.207 | -0.211 | 0.182 | -0.240 | 0.158 | -0.148 | 0.174 |
| **Protein %** | -0.047 | 0.038 | -0.047 | 0.034 | -0.044 | 0.038 | -0.045 | 0.034 | -0.047 | 0.029 | -0.044 | 0.038 | -0.041 | 0.037 |
| **Fertility** | -3.199 | 3.271 | -3.199 | 3.271 | -3.132 | 2.863 | -3.199 | 3.271 | -1.313 | 1.119 | -3.132 | 2.863 | -1.015 | 0.635 |
| **Survival** | -7.192 | 3.200 | -4.298 | 1.859 | -7.192 | 3.200 | -4.298 | 1.859 | -2.221 | 0.796 | -7.192 | 3.200 | -3.954 | 1.064 |
| **SCC** | -8.715 | 20.053 | -7.940 | 20.053 | -8.715 | 18.006 | -7.940 | 20.053 | -1.041 | 1.156 | -8.715 | 18.006 | -2.203 | 2.263 |
| **Mamm Sys** | -5.861 | 2.778 | -5.861 | 2.778 | -3.703 | 2.564 | -5.861 | 2.778 | -3.082 | 1.659 | -3.703 | 2.564 | -2.567 | 1.480 |
