# Supplementary material for: Multibreed genome wide association can improve precision of mapping causative variants underlying milk production in dairy cattle
Source: BMC Genomics. 2014 Jan 24;15:62. doi: 10.1186/1471-2164-15-62 (PMC3905911; doi:10.1186/1471-2164-15-62)
Supplement: Additional file 3: Table S3 — False discovery rates for comparative GWAS at a) P < 10-8 significance and b) a suggestive threshold of P < 10-5. (NS) No significant SNP to test. [file 1471-2164-15-62-S3.doc]

**Additional file 3: Table S3. False discovery rates for comparative GWAS at a) P<10^-8^ significance and b) a suggestive threshold of P<10^-5^. (NS) No significant SNP to test.**

| a) |  |  | **Between Breeds** | | **Within Breeds** | | | |
| --- | --- | --- | --- | --- | --- | --- | --- | --- |
|  | **Trait** | **Multibreed** | **Holsteins** | **Jerseys** | **Hol Bulls** | **Hol Cows** | **Jer Bulls** | **Jer Cows** |
| **Milk Production** | Fat | 3.95E-06 | 2.38E-06 | 1.11E-06 | 1.62E-06 | 3.01E-06 | 6.42E-06 | 7.84E-06 |
|  | Milk | 1.67E-06 | 2.65E-07 | 3.60E-07 | 1.15E-06 | 1.19E-06 | 1.59E-06 | 2.99E-06 |
|  | Protein | 2.35E-06 | 1.88E-07 | 3.00E-07 | 7.24E-07 | 1.92E-06 | 2.43E-06 | 1.57E-06 |
|  | Fat% | 1.60E-06 | 8.22E-07 | 5.21E-07 | 1.85E-06 | 3.03E-06 | 1.24E-06 | 9.34E-06 |
|  | Protein % | 7.71E-07 | 5.21E-07 | 8.50E-07 | 1.44E-06 | 1.87E-06 | 1.16E-06 | 9.13E-06 |
| **Reproduction** | Fertility | NS | 8.54E-05 | NS | NS | 1.66E-04 | NS | NS |
| **Health** | Mam. Syst | 3.16E-04 | 3.82E-06 | 4.65E-05 | NS | 2.81E-06 | NS | 5.27E-04 |
|  | Survival | 2.86E-06 | 1.09E-05 | 8.43E-06 | 7.02E-04 | 9.27E-06 | NS | 2.86E-05 |
|  | SCC | 3.95E-04 | 1.05E-04 | 2.29E-05 | 3.51E-04 | 4.51E-04 | 2.14E-05 | NS |
|  |  |  |  |  |  |  |  |  |
| b) |  |  | **Between Breeds** | | **Within Breeds** | | | |
|  | **Trait** | **Multibreed** | **Holsteins** | **Jerseys** | **Hol Bulls** | **Hol Cows** | **Jer Bulls** | **Jer Cows** |
| **Milk Production** | Fat | 6.83E-04 | 4.99E-04 | 2.28E-04 | 4.93E-04 | 7.73E-04 | 6.17E-04 | 7.40E-04 |
|  | Milk | 4.22E-04 | 9.19E-05 | 1.10E-04 | 2.57E-04 | 2.56E-04 | 3.02E-04 | 3.76E-04 |
|  | Protein | 4.20E-04 | 7.36E-05 | 9.75E-05 | 2.69E-04 | 7.03E-04 | 3.69E-04 | 2.59E-04 |
|  | Fat% | 7.11E-04 | 2.43E-04 | 2.07E-04 | 5.74E-04 | 7.94E-04 | 3.34E-04 | 8.72E-04 |
|  | Protein % | 3.50E-04 | 2.07E-04 | 3.39E-04 | 6.08E-04 | 6.82E-04 | 4.54E-04 | 1.38E-03 |
| **Reproduction** | Fertility | 9.16E-02 | 2.78E-03 | 5.06E-02 | 6.32E-01 | 4.03E-03 | 6.32 | 3.95E-01 |
| **Health** | Mam. Syst | 8.72E-03 | 5.23E-04 | 2.08E-03 | 4.30E-02 | 4.28E-04 | 1.24E-01 | 3.67E-03 |
|  | Survival | 8.99E-04 | 1.61E-03 | 1.01E-03 | 2.87E-02 | 1.79E-03 | 3.07E-02 | 1.47E-03 |
|  | SCC | 1.31E-02 | 3.73E-03 | 1.80E-03 | 9.02E-03 | 8.71E-03 | 1.76E-03 | 2.55E-02 |
