# Supplementary material for: Multibreed genome wide association can improve precision of mapping causative variants underlying milk production in dairy cattle
Source: BMC Genomics. 2014 Jan 24;15:62. doi: 10.1186/1471-2164-15-62 (PMC3905911; doi:10.1186/1471-2164-15-62)
Supplement: Additional file 4: Table S4 — The number of very significant QTL (P < 10-20) identified for milk production traits and significant QTL (P < 10-8) for production and functional traits, within breeds, within genders, and in the multibreed data set. [file 1471-2164-15-62-S4.doc]

**Additional file 4: Table S4.** The number of very significant QTL (P<10^-20^) identified for milk production traits and significant QTL (P<10^-8^) for production and functional traits, within breeds, within genders, and in the multibreed data set.

|  | **Multibreed** | **Holstein** | **Jersey** | **Holstein Cows** | **Holstein Bulls** | **Jersey Cows** | **Jersey Bulls** |
| --- | --- | --- | --- | --- | --- | --- | --- |
| Fat | 3 | 4 | 3 | 2 | 2 | 1 | 0 |
| Protein | 4 | 47 | 22 | 9 | 3 | 0 | 1 |
| Milk | 5 | 9 | 21 | 4 | 5 | 4 | 2 |
| Fat % | 3 | 3 | 11 | 3 | 3 | 3 | 1 |
| Protein % | 8 | 12 | 5 | 10 | 3 | 5 | 1 |
| Fertility | 0 | 0 | 0 | 0 | 0 | 0 | 0 |
| Mamm. Syst. | 0 | 1 | 0 | 0 | 4 | 0 | 0 |
| Survival | 0 | 1 | 4 | 2 | 0 | 5 | 0 |
| SCC | 24 | 1 | 0 | 0 | 1 | 0 | 0 |
