# Supplementary material for: Multibreed genome wide association can improve precision of mapping causative variants underlying milk production in dairy cattle
Source: BMC Genomics. 2014 Jan 24;15:62. doi: 10.1186/1471-2164-15-62 (PMC3905911; doi:10.1186/1471-2164-15-62)

**Additional file 5: Figure S1. Q-Q Plot of significant effects for milk production traits in Holstein and Jersey cattle. (a) The large number of significant SNP in the milk production trait plots are driven by a small number of QTL regions, as demonstrated by re-drawing the plots with BTA6, BTA14, and BTA20 removed from the analysis (b).**


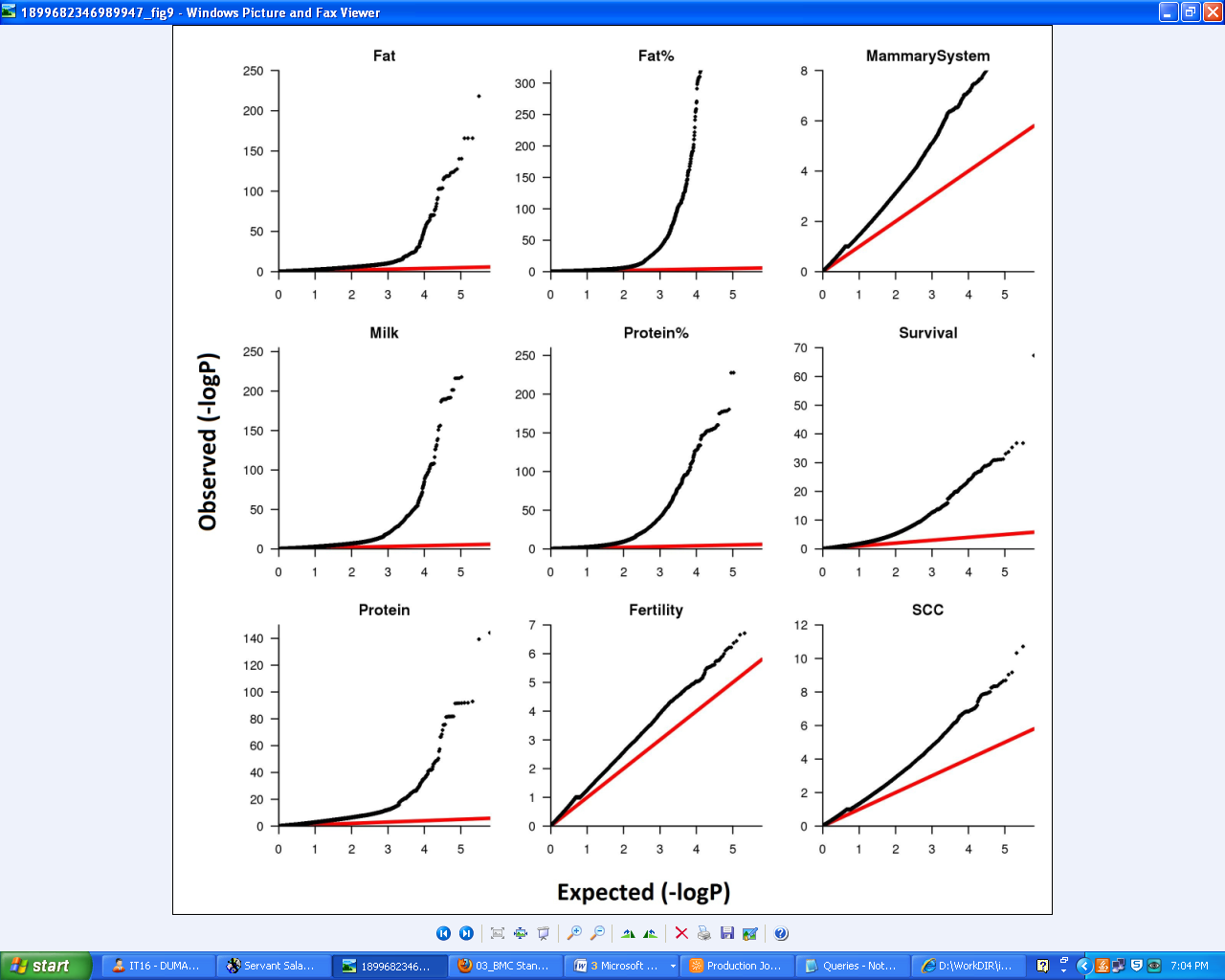

Supplement: Additional file 5: Figure S1 — Q-Q Plot of significant effects for milk production traits in Holstein and Jersey cattle. (a) The large number of significant SNP in the milk production trait plots are driven by a small number of QTL regions, as demonstrated by re-drawing the plots with BTA6, BTA14, and BTA20 removed from the analysis (b). [file 1471-2164-15-62-S5.doc]
