# Supplementary figures and images for: Multibreed genome wide association can improve precision of mapping causative variants underlying milk production in dairy cattle
Source: BMC Genomics. 2014 Jan 24;15:62. doi: 10.1186/1471-2164-15-62 (PMC3905911; doi:10.1186/1471-2164-15-62)

**Additional file 6: SNP map and P-values for multibreed GWAS of 9 milk production traits**.


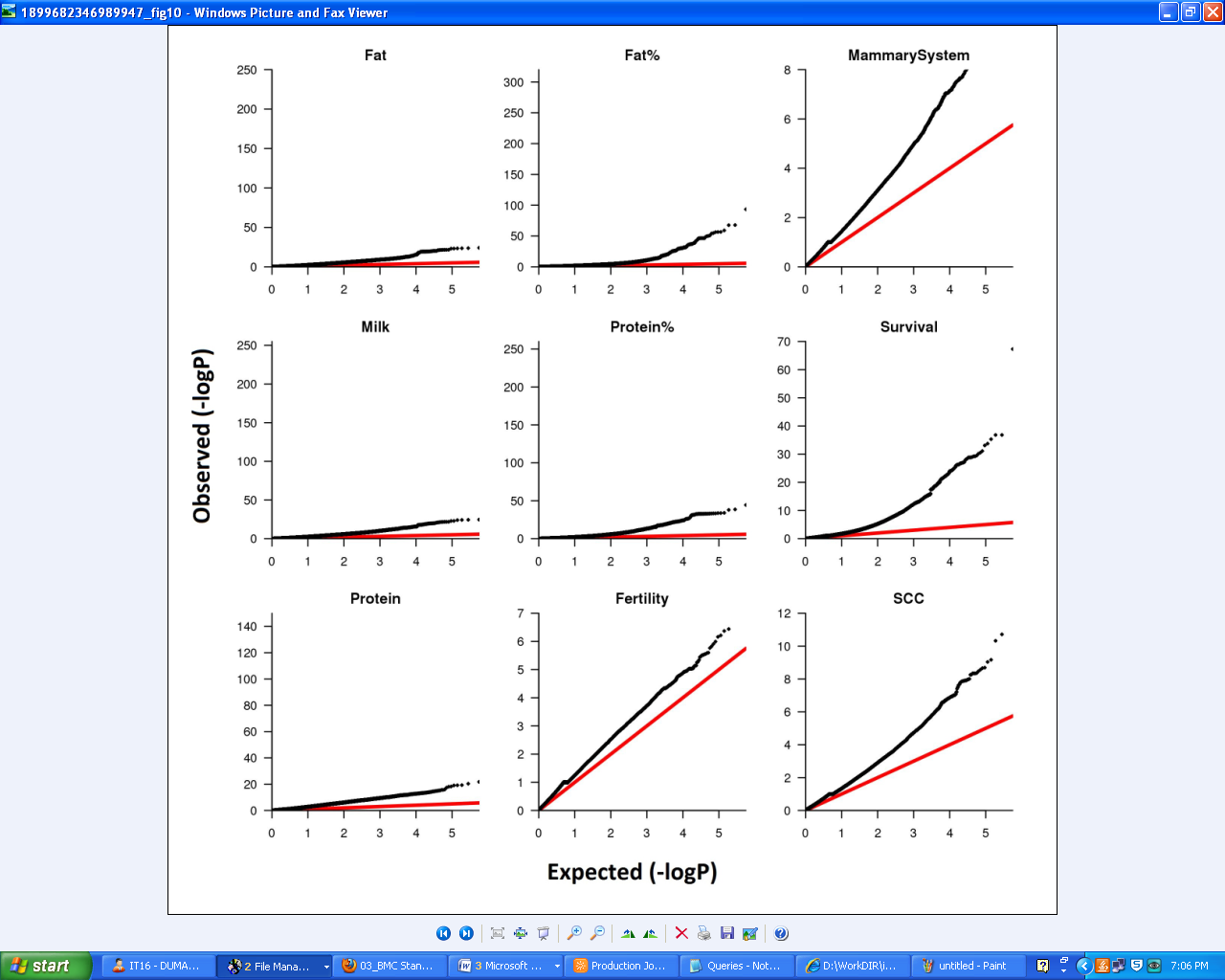

Supplement: Additional file 6 — SNP map and P-values for multibreed GWAS of 9 milk production traits. [file 1471-2164-15-62-S6.doc]
